# School Choice in London and Paris – A Comparison of Middle-class Strategies

**DOI:** 10.1111/spol.12079

**Published:** 2014-05-26

**Authors:** Michaela Benson, Gary Bridge, Deborah Wilson

**Affiliations:** a Department of Sociology, Goldsmiths, University of LondonLondon, UK; b School for Policy Studies, University of BristolBristol, UK; c Centre for Market and Public Organisation and School for Policy Studies, University of BristolBristol, UK

**Keywords:** School choice, London, Paris, Middle-class

## Abstract

Education is one major public service in which quasi-markets and other choice-based mechanisms are now established methods of delivery. The types of school people choose, and the extent to which their choices are realized, have a fundamental impact on the outcomes of any mechanism of school choice. In this article, we provide a comparative analysis of the school choice strategies of middle-class families in London and Paris. We draw on approximately 200 in-depth interviews carried out across the two cities. This enables us to investigate the extent to which middle-class school choice strategies transcend the institutional context provided by both the local (state and private) schools market and national education policy in England and France. We discuss these findings in the context of current school choice policy and consider their implications for future policy design.

## Introduction

Education is one major public service in which choice-based mechanisms are established methods of delivery. Parental choice of school has been part of the English education system since 1988, and choice is similarly well established in countries such as Chile, Sweden and the USA (Allen and Burgess 2010). In 2007, President Sarkozy included the liberalization of the French education system in his election manifesto, which has led to an increase in the degree of choice parents have over the schools attended by their children.

While in theory it has been argued that school choice mechanisms can both improve the performance of, and access to, different schools (Le Grand 2007), in practice the evidence for both is, at best, mixed (Allen and Burgess 2010). The design and evolution of school choice policies (and education systems more generally) have failed to address the perpetuation of inequality and, in particular, the ongoing middle-class advantage in the education field (Greener and Powell 2009; see also Felouzis 2009). Class differences in individuals' roles as ‘consumers’ in user-based mechanisms are key in relation to processes of choice and voice across different public services (Greener 2008; Simmons *et al*. 2009). How and why individuals choose between schools – and the extent to which they are able to realize their choices – has a fundamental impact on the outcomes of any choice-based mechanism.

This article explores these processes as they play out among the urban middle-classes in London and Paris, drawing on in-depth interviews with respondents in both cities. We are able to compare both the basis for, and strategies of, school choice for these parents in two very different institutional and cultural contexts. This comparative research design therefore enables us to investigate the extent to which middle-class school choice strategies are shaped by or, conversely, transcend the context in which they take place, which in turn has implications both for the outcomes and future design of school choice policy.

There is a substantial literature on middle-class engagement with the process of school choice. The theoretical framework for much of this work is drawn from cultural reproduction theory, originating from the work of Pierre Bourdieu (Bourdieu 1984; Bourdieu and Passeron 1977). A central focus is the role of cultural capital, its transmission, acquisition and deployment, as a key mechanism in explaining class differences in strategies over school choice and differences in educational attainment more widely (Di Maggio 1982; Sullivan 2001). The operationalization of cultural capital in this way leads to a distinction between classes in the way that choices are made. Influential studies in this field suggest that middle-class parents are active choosers, that their choices are long term and strategic, and that they mostly choose schools on behalf of their children. In contrast, working-class parents are seen to be more passive and more locally oriented, with their choices being led by their children (Reay and Ball 1998; Reay and Lucey 2003; van Zanten and Obin 2010). In another paper (Bridge and Wilson 2014 forthcoming), two of the current authors discuss this theoretical perspective in more depth, comparing it with an alternative framework for theorizing class dimensions of school choice which is based on the sociological rational choice theory put forward by Boudon (1974; see also Goldthorpe 1996).

The empirical literature for England reveals a range of middle-class choice strategies that include moving house to be closer to good schools, getting involved in local churches to ensure entry to high performing faith schools, pursuing appeals if their child is not admitted to the school of choice, and opting out into the private sector (Ball *et al*. 1995; West and Noden 2003). The pressures of school choice are felt most keenly in London, where the particular combinations of social mix, housing and schools in different neighbourhoods create specific tensions around the choice process (Butler and Hamnett 2010; Butler and Robson 2003; Flatley *et al*. 2001). Evidence of similar strategies has been found in other national contexts (Lund 2008; De Graaf *et al*. 2000; Yoon and Gulson 2010). These strategies relate to sequences of school choices over a child's educational career in ‘circuits of schooling’ (Ball *et al*. 1995). In these circuits of schooling, middle-class children tend to go to ‘better’ schools than their working-class peers, creating the potential for increasing social segregation across schools in cities such as London (Coldron *et al*. 2010).

There is a similar body of literature related to middle-class schooling strategies in France, starting with Ballion's (1982) focus on the middle-classes as consumers of schooling. Such strategies may have elements of school avoidance (François and Poupeau 2004), but can also be related to the desire to access high performing establishments (Oberti *et al*. 2012). Documented strategies include those listed above for England (van Zanten 2011; van Zanten and Obin 2010), as well as using the *demande de dérogation*, a mechanism for opting out of the *carte scolaire*, the residence-based school assignment discussed further below (see also Barrault 2009). As several authors highlight, the more recently introduced system of school choice in France is a source of significant debate (see e.g. van Zanten and Obin 2010; Oberti *et al*. 2012) with clear links made between educational inequality and urban inequality (Oberti 2007; Oberti *et al*. 2012). There is also an emerging body of literature that addresses how families and institutions respond to the recent relaxation of the *carte scolaire* (van Zanten and Obin 2010; Barrault 2011).

There is both qualitative and quantitative evidence that parents choose schools at least partly on the basis of academic standards (Burgess *et al*. 2011). Other factors include the popularity of the school; for example Gibbons and Machin (2003) identify ‘herd’ behaviour in primary school choice where over-subscription becomes significant for parental choice, irrespective of school/pupil performance. Additionally, it becomes clear that social mix or peer group – in terms of social class and ethnicity – is a key factor on which parental choice of school is based, along with other less quantifiable characteristics such as ethos or ‘school climate’, and attention paid to the child (Bagley 1996; Ball *et al*. 1995; Ball 2003; van Zanten 2011). Studies in both England and France have explored middle-class parents actively choosing the local school (Reay *et al*. 2011; Raveaud and van Zanten 2007). Crozier *et al*. (2011) argue that such parents are, perhaps unwittingly, engaged with the discourse of competition for their children's educational success.

Assessments of the reputation as well as the performance of schools are, understandably, part of the process of school choice. In making educational choices parents may identify the social mix within a school, this often becoming a proxy for its quality. Such assessments are often based on impressions, rumours and informal knowledge through social networks: the ‘hot grapevine’ of information (Ball and Vincent 1998; van Zanten 2002; Fack and Grenet 2010). There is less evidence of (middle-class) choosers using the published school performance information to inform their choice in either England or France. Karsten *et al*. (2001) find that middle-class parents in England and France use published performance data, but that there is no conclusive evidence that the publication of this data has a major influence on their choice of school.

Closest to the current article is Raveaud and van Zanten's (2007) comparative analysis of middle-class families in London and Paris who make a deliberate choice to send their children to the local state-funded school (as discussed above, see also Reay *et al*. 2011 for an analysis of similar schooling strategies in different English cities). They argue that the different (national and regional) policy contexts have less influence on choice strategies than do parental values and resources, and that the choices made often depend on the local context in which they take place. As they stress, this includes the available residential and schooling opportunities as well as the concentration of ‘people like us’ and normative schooling trajectories. Our analysis builds on this work but takes a broader perspective on choice – across the full range of state and private schools accessible across different neighbourhoods – in order to investigate middle-class school choice processes in the two global cities of London and Paris. Understanding school choice in this way, and particularly by comparing two policy contexts, helps to identify how strategies may transcend the institutional structures that are often assumed to frame them.

## Background to Study and Methodology

The empirical data on which this article draws was part of a large comparative study, ‘The middle classes in the city: social mix or just “people like us”? A comparison of Paris and London’, which compared the attitudes and practices of middle-class residents living in neighbourhoods that had different degrees of exposure to social mix across Paris and London.^1^ Interviews with up to 350 residents involved discussions of their reasons for moving to the neighbourhood; prior employment and residential histories; attitudes to the neighbourhood and to social mix; daily and weekly routines; practices of social reproduction (including schooling strategies); and political outlooks.

Respondents were from a range of positions within the middle-classes. Our understanding of the middle-classes extends beyond occupation and into the recognition of the wider assets – including property ownership and education – that serve to position people within the middle-classes (see e.g. Savage *et al*. 1995). Included within this sample were people from a range of occupations including lawyers, journalists, financial services workers, teachers, civil servants and self-employed business owners. Most London respondents were owner-occupiers (with or without mortgages), while in Paris, where tenure is more mixed, respondents were either owner-occupiers or private renters. The educational level of our respondents varied generationally, with younger generations of parents being educated to a higher level.

For the current analysis we selected respondents who were parents or carers with school-age children (five to 18) and parents with pre-school-age children anticipating school choice (92/121 interviews in Paris; 120/172 in London). Of further note here is that we deliberately do not attribute school choice practices recalled in this article to particular fractions of the middle-classes – the data was not collected with such fractions in mind and, therefore, any analysis along these lines would be partial.

The analysis was conceptualized primarily in terms of the ‘what’ and ‘how’ of school choice. The ‘what’ question concerned what it was the respondents considered that they were choosing: what aspects of schools or education in general were privileged and what aspects were ignored? The ‘how’ question looked at the choice strategies themselves. These dimensions of school choice were analyzed in the context of the institutional similarities and differences in the education systems between England and France; London and Paris.

## Institutional Context: Key Differences

Here we briefly outline the main elements of school choice policy in England and in France, focussing on the key differences that are relevant to our research questions. For a broader discussion of the policy context in the two countries, see Burgess *et al*. (2007) and van Zanten and Obin (2010) respectively. There are three main considerations to take into account when considering the exercise of school choice (van Zanten and Obin 2010): the location of the school (urban/rural; proximity of others); the degree of openness of officials in allowing movements between districts; the characteristics of the school environment. In these terms, London and Paris may share more similarities than either one does with more rural areas in its own country. Moreover, in both cities, local education authorities (Académies) may have different degrees of discretion in the extent to which they implement national policies and how, which in turn depends on the history and specifics of the local schooling and broader local policy environment. Our focus is a cross-city comparison: we therefore do not focus on such specific differences in the following discussion of the institutional context; rather, our aim is to highlight key differences across the ways school choice policy is manifested in London and in Paris in order to investigate the extent to which such differences affect middle-class schooling strategies.

### Allocation of school places and the extent of parental choice

The *carte scolaire* has been the primary method of school place allocation in France since the early 1960s (see Fack and Grenet 2008 for a discussion of its evolution). This is a policy of residence-based school assignment administered by local education authorities (in the case of secondary education) and local municipalities (in the case of primary education), whereby children are assigned places to their local primary or secondary school within their defined district of residence.

As part of his 2007 election campaign, Sarkozy significantly relaxed the *carte scolaire* through the *demande de dérogation* (van Zanten and Obin 2010; Oberti *et al*. 2012). This is a request to be considered for a school other than the one assigned on the basis of residence, and demonstrates a shift towards a quasi-market in education replete with consumer choice. Such a request must be on the basis of one or more of a list of published, ranked criteria, which include disability or other medical need; financial hardship; particular course of study required; siblings; residence near the border of another district. If there are places available in the requested school, the *demande de dérogation* is taken into account and children can be re-assigned to the school requested. This is the primary means by which parents are currently able to exercise school choice within the state sector in France. What does become clear, as van Zanten (2011) highlights, is that middle-class parents may be better equipped (in terms of cultural capital) to negotiate the *demande de dérogation*. These may also be matched by institutional strategies focussed on attracting middle-class students.

Parental choice of state school is much more established in England, having been introduced as part of the Education Reform Act 1988, which brought in open enrolment, overlapping catchment areas and per capita funding. Parents specify and rank their preferred schools, and places are allocated on the basis of those preferences if capacity permits. The range of types of school from which parents can choose is wider than in France, and is continuing to grow with the current UK government's policy of encouraging diversity of provision. There are differences in admissions criteria both across local education authorities and across certain types of school (e.g. religious schools), and how this impacts on local school ‘markets’ continues to be a particular issue (Allen and West 2009; Flatley *et al*. 2001). If demand exceeds a school's capacity, there is a published list of criteria by which places are allocated. These vary across local authorities, but admissions criteria commonly include looked-after children, special educational needs, having a sibling already at the school, with residential proximity to the school often employed as a final, ‘tie-break’ device when schools are over-subscribed (Burgess *et al*. 2011). West *et al*. (2009, 2011) provide a detailed account of English secondary schools' admissions criteria and practice in light of changes to the regulatory context. A few local authorities have introduced lotteries as an alternative tie-break mechanism (Allen *et al*. 2013) in an attempt to break the school–home link and therefore, in theory, the extent to which the middle-class can gain advantage via the housing market. The priority given to residence within the school-place allocation mechanism is, therefore, fundamentally different across the two systems. The extent to which this difference impacts on middle-class school choice strategies (via the housing market) is discussed below.

### Public reporting of school performance

Performance tables are produced annually for every state school in England by the Department for Education and have been since 1992 and 1997 for secondary and primary schools respectively. A wide range of indicators are published, including various measures of average test scores; progress and/or value added measures; unauthorized absences (Wilson and Piebalga 2008). They are widely reported in the national and local media: individual schools are ranked on the basis of their outcomes, with attention often focused on the ‘headline’ figures of variants of the proportion of students achieving at least five good GCSE passes or equivalent (the minimum requirement to enter post-compulsory education at age 16). The school league tables are aimed at informing parental choice, as well as being employed as part of a concurrent system of bureaucratic accountability (Wilson 2011). Further information and accountability is provided by a system of regular school inspections carried out by Ofsted, the schools regulator, whose gradings and reports are publicly available.

School performance information about lycée (which take students from 15 to 18 years old) is publicly available in France through the central government examinations portal (http://www.franceexamen.com [accessed 27 April 2013]), which publishes the *Baccalauréat* results (a national, standardized qualification taken at age 18). Rankings also appear in the public press (e.g. *Le Monde de l'Education*, *L'Express*). Official information about educational institutions prior to lycée is not publicly available and there is no equivalent of Ofsted in France. Even at lycée level, it is apparent that the provision of information to assist school choice is not an official objective of rankings; rather, performance indicators were introduced within the existing hierarchical administrative culture (Karsten *et al*. 2001). These assessments are not wide-ranging as in the English case, but the information made publicly available about lycée in France does, however, contain value-added measurements and comments from researchers and other experts. These measures locate the student population in terms of their age and social backgrounds, indicating the numbers of students in each school who started the course and finished it, as well as the proportion of students passing the exams. There is more emphasis on, and public awareness of, school performance data in England compared to France.

### The relationship between the state and private school sector

In Britain, private schools are autonomous from the state. They often command high fees (see below), and hence are only accessible to fractions of the population with the means to meet these. Although they are often perceived as a realm of the elite, Walford (1990) stresses that these perceptions neglect a detailed knowledge of a diverse sector, but argues that in Britain its presence has exacerbated the perpetuation of inequality in schooling.

There is a much closer relationship between the private and state sector in France. In France, the historical relationship between the two sectors has been heavily influenced by the state/church conflict in education and the secular state system: 95 per cent of French private schools are Catholic (van Zanten 2011). The vast majority of private schools are under contract with the state: the state controls the number of schools, appoints teachers, pays their salaries and contributes a large proportion of the running costs. Private schools have to follow the national curriculum and can only charge for education that goes beyond what the state would ordinarily provide (e.g. religious instruction). This results in relatively low fees for private education in France: for private schools under contract to the state (98 per cent of all private schools), fees range from €500 to €1,500 per year. (The fees of the 2 per cent not under contract with the state range from €4,500 to €7,800 [CIDE 84 2013].) English private school fees are, on average, much more expensive and approached £10,000 per year in 2008, although that average masks significant regional differences (Blundell *et al*. 2010). The difference in costs is reflected in differences in the proportions (as well as in the potential diversity of pupils) attending private schools in the two countries: approximately 7 per cent of children in England are educated privately, rising to 10 per cent in London and the South East (Blundell *et al*. 2010), while in France, attendance at private schools is noticeably higher (14 per cent at primary, 20 per cent at secondary [van Zanten and Obin 2010]). Such differences may partly also reflect additional admissions criteria for entry to English private schools beyond the ability to meet the economic costs – attainment in entrance exams, religion, for example – that act as additional admissions constraints.

While heavily state regulated, French private schools are not subject to the *carte scolaire*, so there is no residence-based allocation criterion for places at private schools in either England or France. Private school choice can, therefore, run parallel with the process of state school choice in both countries, as we discuss below.

## School Choice in London and Paris

In this section we describe how, given the different institutional contexts in Paris and London, school choice takes place among the middle-class in these cities. It examines what people want from a school, the information they use to inform that decision, and the strategies they employ to achieve their desired outcome. Our aim is to provide an overview of different practices and relate these to wider questions of school choice policy.

### What middle-class parents want

By far the most common basis for choice of school, in London and in Paris, is social mix: the school peer group. Respondent P04 lived in Peckham, a socially mixed neighbourhood in London. She was in her 30s, with two children of primary school age, and explained that the issue of social mix was a significant source of anxiety for parents looking to educate their children in the city:

*‘… obviously, the thing that is a big issue for parents in London is school … that is where it all starts to hit the fan in terms of social mix and politics and culture and everything.’* (P04, London)

Getting the ‘right’ social mix heavily influences schooling strategies, and our interview data reveal a common story focussed on peer group, described in terms of both social class and ethnicity. There is a common concern about both the prospective school population and parents' perceptions of what other ‘people like us’ do when educating their children and choosing schools. In this respect, peer groups at the level of children are nested within the peer groups of parents, demonstrating the continuing concern over social reproduction.

Understanding of the social mix within schools lead to strategies aimed at avoidance. LR10, who had two school-age children, explained how she had chosen to rent property in Le Raincy because of its good publicly-funded schools, even though she did not like the overall bourgeois feel of the neighbourhood. She worked in retail while her partner trained people in photography:

*‘I did not want to find myself in a neighbourhood that was too sensitive and where my child could have bad company, too much bad company at school … “Dubois” appears to have better company I would say.’* (LR 10, Paris)^2^

B06, a researcher (her partner was an independent financial adviser) and who had two primary school-age children, explained the basis of these perceptions:

*‘… it's not that these schools aren't good, but the perception is that there are lots of coloured people there so we don't want to send our children.’* (B06, London)

On the other hand, however, some respondents elect a particular school on the basis of its particular social mix. BL05, whose children had been to school in the area and who was a teacher explained:

*‘I suppose “Walkers” catchment doesn't actually include any council estates, “Anderson”… a little one, but I think the majority of children are middle-class.’* (BL05, London)

In London, both social and ethnic mix feature prominently in the discussion of the qualities of local state education, from discussions of the high council (social housing) estate intake of one local secondary school to reflections on the number of minority ethnic pupils leaving the school grounds at the end of the day. For the large part, there are concerns about how such mix might impact on a child's educational career, particularly at secondary level, with social and ethnic mix often serving as a proxy for quality of education in the school – we return to this in the following section. There is, however, an additional sense that over time, as more middle-class people move into areas, and if there is a collective middle-class move towards educating children in the state sector, then the quality of schools will improve to the point where they can consider educating their children there, demonstrating the importance of ‘people like us’ in the schooling choices of the London middle-classes, a focus that was not so evident among Paris respondents.

In Paris, there were similar discourses about social and ethnic mix that led to school avoidance. A strong discourse that schools wholly funded by the state had the wrong type of social mix seemed to underline such discourses. These claims were particularly intense in the gentrifying, socially and ethnically mixed neighbourhood, with an overall consensus that one of the major problems with educational supply in the area is that middle-class families have been opting out of state education, leaving a ‘white minority’.

On the other hand, however, in Paris there was also a sense that social mix within schools was good for children. The valorization of social mix and mixing was as common as the discourse about the need to escape particular types of social mix in a way that was not matched by our respondents in London (although see Reay *et al*. 2011). 9E-03, an air stewardess married to a pilot who had three children, two of secondary school age and one in the last year of primary school, emphasized this point. She explained their choice between two publicly-funded secondary schools in the neighbourhood, highlighting how they had deliberately selected the one that was known for being more mixed and less elite than the other:

*‘ “Martin” is all reputation, it is very selective, it is very bourgeois etc. And “Bernard”, has more social mix, and I even made the choice to send him [son] to “Bernard”… he would have been more protected at “Martin”, but it had a degree of selectivity that bothered me.’* (9E-03, Paris)

Respondents who favoured social mix believed that by educating their children in an environment such as this they would open their minds and give them a sense of the real world. As Raveaud and van Zanten (2007) argue, the decision of French parents to educate their children in local schools needs to be understood within the context of French citizenship, in particular the duty to avoid social segregation and inequality. Nevertheless, such choices, while underpinned by a sense of Republican egalitarianism, can have perhaps unintended consequences, as collective action aimed at the improvement of schools is underwritten by middle-class valuations of what constitutes a good educational environment. This can result in segregation within schools and their further colonization by the middle-classes (van Zanten 2002).

Overall, the primary middle-class discourse in both London and Paris regarding the basis for school choice centres on social mix; on getting the ‘right’ school peer group for one's child. As we now discuss, this is fundamentally linked with both the perceptions of the parents' own peer group and perceptions of school ‘quality’.

### Sources of information for school choice

In both cities, the information that respondents use to make their choices about schooling are drawn mostly from informal sources of knowledge: their own experiences, the opinions of teachers and the opinions of other middle-class parents. What is clear is that the publicly available performance criteria are not the main source of information for school choice among parents in either city; rather they focus more on the visible aspects of schools, and in particular the social and ethnic characteristics of the school population, making these the basis of their judgements of what the school offers (van Zanten 2009).

The experiences that they draw on to make these decisions range from their own educational experiences and the desire to reproduce these for their children, to first-hand observations at the school gates. In both Paris and London there are examples of people making choices about their children's education based on their own educational experiences. 9E-03 explained how she had chosen to send her children to the same primary school that she had attended:

*‘… we reproduce the pattern. We all stayed here … Me, I was at “Durand”, I have three children, they all three went to “Durand” … It is very reassuring in fact.’* (9E-03, Paris)

In some of the London neighbourhoods, respondents also stressed this continuity with their own education experiences. WH19, on maternity leave from a government agency, stressed that she had sent her three-year-old son to a private nursery precisely to reproduce the opportunities that she had had:

*‘[Sending me to private school] is something my parents did for me and I always felt that because they did that for me I wanted to do it for my children.’* (WH19, London)

First-hand observations of the behaviour of students, combined with gathering information from teachers, family and other middle-class parents commonly form the basis of judgements that lead to avoidance of particular schools. In making choices over schooling, respondents rely in large part on a ‘hot grapevine’ of information (Ball and Vincent 1998) circulating between middle-class families and within social networks.

It was clear that rumours about schools circulating in these ways informed opinions about whether particular schools were suitable, at times even long before such choices needed to be made:

*‘I know there have been discussions between my wife and my in-laws in relation to the high school on Avenue “Simon”, because my mother-in-law finds it a bit of a dodgy place. I do not think that high school is particularly worse than elsewhere, but there is a controversy in the family over whether to send Gabrielle over there.’* (9E-19, commissioner in a museum/furniture restorer, one daughter, aged five, Paris)

*‘I'm basing this on absolutely no real information* this is all sort of middle-class rumours *and stuff, it's a rough school. So I don't know what we'll do …’* (P04, stay-at-home mother/journalist, two primary school-age children, London, emphasis added)

In contrast, there is seemingly very little reliance on publicly available performance criteria in making schooling choices. In some cases, respondents demonstrated an awareness of this data, for example the high ranking of particular schools in both Paris and London. However, in relation to negative assessments of schools in particular, it was often the case that respondents lacked any official knowledge of the standing of the school. This was made particularly evident in an example from a gentrified London neighbourhood, where there was a strong discourse among middle-class residents about avoidance of a local state secondary school. Respondents demonstrated very little knowledge of the Ofsted rating of the school despite the large banner at the school gates announcing the ‘outstanding’ grade it had achieved in its 2009 inspection. This was made clear in a number of interviews, with the analysis demonstrating that respondents largely concentrated on their perceptions of the children entering and exiting the school gates. B30, who worked in financial services and who had a pre-school-age child explained:

*‘… there's a place called “Richards” … which is the local state secondary. Apparently it doesn't have enough students from the area so they come from four or five miles away, but not because it's particularly good … But the results are not great …’* (the school attained 100 per cent A*-C in 2012)

There was also a connection between a school's composition and respondents' perception of its quality, with the school's social and ethnic mix being used by respondents as a proxy for the quality of education provided, sometimes – as above – despite fairly obvious evidence to the contrary. Indeed, in the outer London neighbourhoods, respondents sending their children to state schools stressed that parents and children were largely ‘people like them’, a claim that implicitly denoted the quality of the school.

Consideration of peer group forms the basis of both positive and negative assessments of the quality of state schools by middle-class parents. This is fundamentally linked with the centrality of social mix as a basis for choice, and may in part explain the lack of use of potentially conflicting published school performance data.

### Schooling strategies

In spite of the fundamentally different priority given to residence in the two systems (primary criterion in France; tie-break device in England), residential choice features prominently in middle-class schooling strategies in both Paris and London. However, the reasons for this are different. In London, there is significant pressure on places in a relatively small number of popular, over-subscribed state schools, and this can have a significant impact on property prices (Gibbons and Machin 2003). Moving house to be close to such a state school makes sense if it is financially viable, given the common reliance on proximity as a tie-break device.

In Paris, it is clear that the resonance of residence-based school allocation that is central to the *carte scolaire* influences school choice, in addition to a significant valuation of the local embedded within citizenship ideals (Raveaud and van Zanten 2007). Local state schools have to be deemed the right ones, however, both in terms of the school itself and its role in feeding to other schools in the development of an educational career. For example, CF14 who had three children aged 17, 15 and 10, and so had recent and ongoing experience of different transition points in education, explained their knowledge of this mechanism:

*‘… the location of Chateaufort within the carte scolaire is an attraction to living in the area as not only does it mean that children have access to the “Laurant”, this in turn opens the doors for them to get their children into lycée in Versailles, which includes a lycée classed as the third best in France.’* (CF14, real estate investor/graphic designer)

Residential moves for schools are also a prominent element of the schooling strategies in London:

*‘I mean I was sort of drawing the one-mile diameter around these good schools and then, you know, getting serious with the Google Maps’* (BL07, two children, primary school age, computer programmer/art historian, London).

These individual choices are contextualized as part of a wider process of school selection via residential moves that are cumulative in effect:

*‘… lots more young families moved … in the last ten years, because the primary schools in the area are so good – have a really good reputation. It's a massive attraction … we're constantly getting flyers through from estate agents saying a young family with two children desperately want to move into the area – are you interested?’* (BL17, two children – one primary, one secondary – part-time teacher and TV sports presenter, London)

Importantly, the link between residential and educational choice involves not only moving into neighbourhoods with an attractive educational offer, but also moving out of neighbourhoods as a mechanism of school avoidance. This tends to happen as a result of people moving to a neighbourhood before parenthood when schools were not a criterion of residential choice, or because of the perceived risks of secondary schools for parents with children coming up to secondary school age. Such avoidance strategies can be seen in both Paris and London, often tied to the perceived quality of local secondary schools. In these cases, as described above, social mix often operates as a proxy for the quality of the school, articulated through concerns over how that would impact on their child's peer group and educational experience:

*‘… the idea to leave [20eme Arrondissement] was to find her a public school in an area where the social environment was relatively protected.’* (LR07, four children – one of school age currently enrolled in lycée – stay-at-home parent (ex-information engineer)/executive officer for a national company, Paris)

*‘I'd like to think we'll do the same thing that we did and use the local state school but … you know I don't know what it's going to be like, there's some very [sighs] rough schools here.’* (P04, two primary school-age children, stay-at-home parent/journalist, London)

So despite the very different priority given to residential proximity in the English and French student allocation mechanisms, residential choice figures prominently among our respondents in both London and Paris. There is also evidence of a growing choice-like element in the greater use of appeals against the simple neighbourhood allocation in Paris, for example one respondent submitted a *demande de dérogation* explaining to the interviewer that the school the respondent's child had been allocated to had a high proportion of students from local social housing.

The second common strategy that respondents in both cities adopt is to opt out of state education, selecting private education as an alternative. This was more evident in London than in Paris. Private schooling among respondents in London was often presented as an alternative to moving house (private schools in the UK are not strictly catchment area related), with some respondents discussing weighing up buying a more expensive house versus school fees. These factors are not necessarily mutually exclusive, however. There is also some evidence of a link between house moves and private schools, as some parents seek to get closer to feeder preparatory schools for well-known, high achieving private schools, or to help capture a place in over-subscribed private schools:

*‘… people here actually work backwards … so “Harrison” send forty-six percent of their girls to Oxford or Cambridge, so that's really important to you, your first thing is you want to get them into “Harrison” Girls. So then they look at the feeder school and say, which school has the highest success of getting their girls into “Harrison” Girls?’* (B20, three primary school-age children, accountant/works in the jewellery trade, London)

The range of attitudes to private schooling amongst the London respondents broke down into four categories. There are those who see private schooling as the ‘natural’ or default choice, from the perspective of the choosers themselves:

*‘so we know there were a lot of good private schools round here, and I guess we just defaulted or drifted into the independent system’* (WH13, one primary school-age child, two post-school-age children from a previous relationship who had been privately educated, business owner/housewife, London)

A second group see private education as an insurance policy if they are unable to secure a place at a good state school:

*‘[looked round state schools] one fantastic one, one horrendous one, I'm pretty sure we'd get the spot in the horrendous one and so we have put a deposit down for her to go privately.’* (B37, two pre-school-age children, lawyer/lawyer, London)

Third, in London, where the cost of private schooling is significant, at an average of £14,000 per year for a secondary school (Blundell *et al*. 2010), there is also evidence of real compromises and tradeoffs being made (in terms of housing space, or financial commitments) for a section of the London respondents:

*‘… he's 12 now and he's been there [independent school] since he was seven. So five years he's been there … we have one car, we don't have a holiday … things are a bit tight. I have two lodgers normally … to obviously contribute to his school fees, and I don't know that I would necessarily do that.’* (BL35, two secondary school-age child, counsellor/IT services consultant, London)

A final set of responses, the only theme common across the two cities, suggest ideological conflict over private schooling but that it is the children's perceived welfare that prevails:

*‘… when you look at the middle school and high school in “Noisy”, this social mix no longer really exists, the kids all come from practically the same neighborhood, they are all from the same social background, many of the kids are the children of immigrants … that's been a heartbreak for me to have to put my kids in private school, because that is not my idea of how things should be’* (N25, two secondary school-age children, civil servant, Paris).

Among respondents in Paris there is much more resistance to private schooling than in London, accompanied by significant support for state schools, for example:

*‘To send her to private school is against my principles’* (9E-19, one child (aged five), museum commissioner/furniture restorer, Paris)

However, it is also clear that especially in the gentrifying neighbourhood where the social mix is greater than in other neighbourhoods, respondents felt that they had no alternative but to educate their children in local private schools. This choice is very clearly framed by an institutional context that privileges residence-based allocation. Nevertheless, for respondents in both cities, dilemmas over whether to choose state or private education were evident and accompanied by significant anxiety.

*‘… ideologically, I'm quite left-wing, I'd much prefer him to go to a state school than a private school but in the end I can't put my political conscience ahead of his educational needs.’* (B30, one pre-school-age child, financial services worker, London)

*‘… everybody wants to talk about it because it creates such anxiety, all around us there are a lot of people on the left, most people are not comfortable thinking about middle school because they don't want to engage in any kind of avoidance and yet when it's your kid and your kid's education which is at stake, well, you say to yourself okay, maybe it's time to put a bag over your ideology, you know, it's complicated for everybody.’* (N01, three school-age children, regional executive for a national company/researcher, Paris)

## Discussion and Conclusions

The purpose of this analysis is to investigate middle-class processes of school choice in two cities which are framed by very different institutional contexts. In London, school choice is well established in the state sector, with residential proximity used as a tie-break device for over-subscribed schools. There is a ready source of official data on a range of school performance indicators and a political culture of encouraging transparency in order to further aid user choice. The private sector is largely autonomous, and private school choice can run parallel to that among state schools. The system in Paris uses residence as its primary criterion, with an element of choice recently introduced into the state sector via the ability to challenge residence-based allocation. Choice via opting into the private sector is more long-standing, although there is a very different historical relationship between state and private schooling in France than in England.

Some contrasts in the middle-class school choice processes in London and Paris can be explained by these institutional contexts. The differences in attitude to private school choice can, at least in part, be traced back to the different relationships between the state and private sectors in the two countries. There is some difference in the discourse on social mix, with more valorization of social mixing via schools in Paris than in London. Lastly, the relative lack of intensity in the discussions of the choice process from our Paris respondents may reflect a less well-established and less ‘heightened’ choice-system than that found in London, in which case we may expect intensity to increase with time as parental choice becomes more embedded in France.

Our findings reveal fundamental similarities across the two cities, however, which transcend the different institutional contexts in which school choice is taking place. First, getting the ‘right’ peer group is central to what middle-class parents are looking for in a school, and being seen to do so by one's own peer group is relatedly important. The composition of a school – in terms of social class and ethnicity – forms a key basis for choice. Moreover, social mix and the perception of ‘quality’ are inextricably linked. Middle-class parents use a school's class and/or ethnic composition as a proxy for the quality of education provided by the school, sometimes despite evidence to the contrary provided by little-used official statistics. In this way, social mix becomes a self-justifying prophecy: parents choose schools on that basis as well as employing it as a proxy for the quality of school to justify their choice.

Second, the strategies employed to realize these choices are similar across both contexts. Despite its very different place in the allocation criteria, proximity is central to both systems, and residential choice is used in order to be close to schools of choice and thus secure a place. We find evidence of moving house both between and within neighbourhoods, for accessing private as well as state schools in both London and Paris. Moving house and opting out of the state sector are not mutually exclusive strategies according to our analysis.

These findings largely confirm those of previous studies into the engagement of middle-class parents with the process of school choice. Peer group forms a large element of the basis for their choice of school, and there are fundamental links between the ‘market for schools’ and the housing market. Our comparative research design enables us to provide additional evidence on the similarities in middle-class process of choice; similarities that transcend very different institutional contexts. In both London and Paris, middle-class parents are strategic and active choosers, and continue to be privileged in the education field because of their ability to access certain (state and private) schools – and, in turn, a certain type of peer group – using their comparative advantage in the housing market.

Our comparative findings have several implications for the outcomes and therefore future design of school choice policy. First, for choice to create the incentive for schools to improve their performance, parents need to choose schools at least partly on that basis. Parents having (a certain type of) peer group as the basis for choice works against the potential for choice as a lever for true performance improvement. The inextricable links between social mix and the perception of ‘quality’ we find in our data suggest this is an ongoing problem, which in turn suggests that the current UK government's focus on transparency and the provision of more data as a means to lever improvements in school performance via parental choice may not in fact drive up standards.

Second, for choice to improve equality of access, the link between the school market and the housing market needs to be broken. The use of residential choice by the middle-classes to achieve their first choice school in our data – despite the very different priorities given to residence in the two systems – works against the potential for choice as a lever for improving equality of access. Attempts at breaking this school–home link in England, via the introduction of lotteries as a tie-break device for example have, however, proved politically very difficult (Allen *et al*. 2013).

More generally, our results point to strong continuities in middle-class practices towards social enclosure in education, despite the differing institutional contexts, incentives and cultural backgrounds to education in London and Paris. To achieve more egalitarian outcomes in the future, policy interventions need to be more focused on considering possible incentive structures that might operate explicitly against these consistent forms of enclavism by middle-class parents. The success of the middle-classes in gaining advantage in the system is also the reason, however, why such interventions may be difficult to implement or sustain.

## Notes

1 The study was a collaborative project funded by ESRC/ANR between partners in France and England, the project team comprising nine researchers (five in France, four in England). Interviews were conducted by French speakers in Paris and English speakers in London between 2010 and 2012 in line with a pre-agreed interview guide. Quotations from French have been translated for inclusion in this article. Benson and Bridge were members of the original project team.

2 We do not report the real names of the schools to preserve anonymity.
